# Increased tolerance to commonly used antibiotics in a Pseudomonas aeruginosa ex vivo porcine keratitis model

**DOI:** 10.1099/mic.0.001459

**Published:** 2024-05-13

**Authors:** Katarzyna Okurowska, Peter N. Monk, Esther Karunakaran

**Affiliations:** 1 Department of Chemical and Biological Engineering, University of Sheffield, Sheffield S10 2TN, UK; 2 National Institute for Health and Care Research, University of Leeds, Leeds LS2 9JT, UK; 3 Department of Infection, Immunity and Cardiovascular Disease, University of Sheffield, Sheffield S10 2TN, UK

**Keywords:** antibiotic susceptibility, ciprofloxacin, *ex vivo* keratitis, gentamicin, meropenem, *Pseudomonas aeruginosa*, ex vivo porcine cornea model

## Abstract

**Introduction**. Bacterial keratitis, particularly caused by *Pseudomonas aeruginosa*, is challenging to treat because of multi-drug tolerance, often associated with the formation of biofilms. Antibiotics in development are typically evaluated against planktonic bacteria in a culture medium, which may not accurately represent the complexity of infections *in vivo*.

**Hypothesis/Gap Statement.** Developing a reliable, economic *ex vivo* keratitis model that replicates some complexity of tissue infections could facilitate a deeper understanding of antibiotic efficacy, thus aiding in the optimization of treatment strategies for bacterial keratitis.

**Methodology**. Here we investigated the efficacy of three commonly used antibiotics (gentamicin, ciprofloxacin and meropenem) against *Pseudomonas aeruginosa* cytotoxic strain PA14 and invasive strain PA01 using an *ex vivo* porcine keratitis model.

**Results**. Both strains of *P. aeruginosa* were susceptible to the MIC of the three tested antibiotics. However, significantly higher concentrations were necessary to inhibit bacterial growth in the minimum biofilm eradication concentration (MBEC) assay, with both strains tolerating concentrations greater than 512 mg l^−1^ of meropenem. When MIC and higher concentrations than MBEC (1024 mg l^−1^) of antibiotics were applied, ciprofloxacin exhibited the highest potency against both *P. aeruginosa* strains, followed by meropenem, while gentamicin showed the least potency. Despite this, none of the antibiotic concentrations used effectively cleared the infection, even after 18 h of continuous exposure.

**Conclusions.** Further exploration of antibiotic concentrations and aligning dosing with clinical studies to validate the model is needed. Nonetheless, our *ex vivo* porcine keratitis model could be a valuable tool for assessing antibiotic efficacy.

## Data Summary

The authors confirm all supporting data and protocols have been provided within the article or through supplementary data files.

## Introduction

Bacterial keratitis poses a global threat to vision, affecting 1.5 to 2 million individuals each year [1,3], and remains underreported in many regions [4]. Among many pathogens associated with bacterial keratitis, *Pseudomonas aeruginosa* infections stand out for their challenging treatment and sight-threatening complications, particularly in developing countries [5]. Multi-drug tolerance often associated with the formation of biofilms by *P. aeruginosa* further complicates treatment, often leading to unfavourable clinical outcomes [6,10]. While some clinical isolates in developed countries, such as the UK, generally exhibit susceptibility to aminoglycosides, fluoroquinolones and meropenem [11], the prevalence of multi-drug-tolerant strains underscores the need for novel therapeutic approaches worldwide [5,7, 9, 12, 13].

Prompt administration of antibiotics early is critical in bacterial keratitis, typically employing broad-spectrum antibiotics to prevent vision loss [7,14]. However, treatment regimens for *Pseudomonas* keratitis vary, including monotherapy or combination therapy with fluoroquinolones, aminoglycosides, cephalosporins and carbapenems [7,9, 15]. Empirical treatment may not suffice, necessitating evidence-based prescriptions guided by antibiotic sensitivity testing [9,16,19]. Notably, such data, typically based on systemic infections, may not fully apply to topically applied ocular antibiotics [20,21].

Current antibiotic testing relies on the MIC, serving as a baseline for determining the potency of antibiotics against bacterial pathogens [9,20, 22, 23]. However, MIC values against planktonic bacteria may not accurately reflect their efficacy on infected tissue, especially when the bacteria are drug-tolerant or the biofilm is present [20,21, 24]. Therefore, MIC value is often analysed together with pharmacokinetic parameters before identifying the line of treatment, usually resulting in using much higher than MIC doses of the antibiotic to achieve therapeutic success *in vivo* [23]. Moreover, antimicrobial resistance mechanisms often further complicate treatment efficacy [25,26], encompassing diverse strategies such as altering antibiotic entry into bacterial cells, modifying antibiotic targets, employing efflux pumps, and enzymatic neutralization of antibiotics [27]. Additionally, biofilm, if present, is known to impede the penetration of antibiotics, slow down the growth of bacteria, change the phenotype and neutralize antibiotics [28,31], thus preventing bacteria from antibiotic-mediated killing. A recent study found that *P. aeruginosa* diversifies after host-cell invasion demonstrating persistence and ofloxacin tolerance independent of the biofilm [32].

The effectiveness of the treatment depends on various factors, including host factors, the virulence of the infecting bacteria, antibiotic type and concentration, epithelial defects, exposure time, drug penetration and infection duration [7,20, 21, 23]. Additionally, topical drug availability in corneas is influenced by numerous factors, such as protein binding, molecular weight, pH, the release of ions and dilution in tear film [24].

In this study, we determined MIC for selected antibiotics to assess the lowest concentration that will inhibit the growth of bacteria, and then we established minimal biofilm eradication concentration (MBEC), accounting for potential antimicrobial tolerance or biofilm formation in our *ex vivo* keratitis model.

Given the limitations of current *in vitro* and *in vivo* models [33], we utilized a previously established *ex vivo* porcine keratitis model [34] to assess selected antibiotic activity. Previously, we demonstrated the optimization of wounding and successfully established infection protocol. During this process, we observed that *ex vivo* corneas swell during storage in media, likely because of the diminished pumping capability of the endothelial cells post-mortem [35]. However, our investigation into reducing swelling through the addition of dextran revealed that it did not significantly affect the infection outcome [34]. Consequently, we discontinued the use of dextran in our experiments. Additionally, in this study, we demonstrated the effectiveness of our glass mould in retaining added solutions of bacteria and drugs. The mould ensures that only the central part of the cornea, measuring 10 mm in diameter, is exposed to the bacteria or drug solution [34]. During the treatment phase, the corneas were exposed to PBS, like in studies *in vivo* on mice [36], with potential future applications using artificial tears. Our methodology here involved further optimizing the experimental protocol and validating the model’s reproducibility. Importantly, the ethical use of animal eyes sourced from abattoirs for research purposes mitigates the need for additional animal sacrifice.

We infected corneas with *P. aeruginosa* reference strains (PA14 and PA01), known for their biofilm-formation capabilities [30,37]. These isolates fall into distinct phylogenetic groups: group 1, comprising the invasive strain PA01, and group 2, including the cytotoxic strain PA14 [38]. Each phylogenetic group is associated with different effects on host cells [39] and clinical outcomes [36,40]. PA01, identified as a moderately virulent strain, forms well-structured biofilms on solid surfaces [41,42] while PA14, highly virulent and more cytotoxic, forms a less structured biofilm [43,45]. Additionally, strain PA01 can penetrate corneal cells and replicate inside, while strain PA14 remains external, which can impact treatment efficacy [36,46]. Our objective in using these distinct strains was to discern potential variations in their behaviour during different stages of *ex vivo* infection and following treatment with established antibiotics. And finally, we selected ciprofloxacin, gentamicin and meropenem to test on our *ex vivo* keratitis model, as examples of clinically relevant antibiotics to which our *P. aeruginosa* strains were classified as sensitive by standard planktonic MIC testing. The results demonstrated an antibiotic tolerance at much higher than MIC and MBEC concentrations in the *ex vivo* keratitis model, despite continuous drug application. Through our study, we demonstrate the versatility of our *ex vivo* porcine keratitis model as a tool for rapidly assessing the effectiveness of antibiotics for ocular infections, providing a cost-effective alternative for further *in vivo* validation. This approach could contribute to the strategic selection of therapeutics with a higher likelihood of success in subsequent *in vivo* studies.

## Methods

### Bacterial strain used

Two wild-type strains of *Pseudomonas aeruginosa* (invasive PA01 and cytotoxic PA14) were a kind gift from Professor Urs Jenal, University of Basel, Switzerland. Both strains were used to infect *ex vivo* porcine corneas and to establish MIC and MBEC values.

### MIC assay

MIC values for *P. aeruginosa* PA01 and PA14 were determined according to the European Committee on Antimicrobial Susceptibility Testing (EUCAST) guidelines [47,48]. The bacterial strains were inoculated in Mueller–Hinton cation-adjusted broth (MHB) for 24 h at 37 °C with agitation at 110 r.p.m. MHB media were chosen as indicated in the EUCAST guidelines [49]. Before each experiment, 0.01 ml of sixfold dilutions of the inoculum were spot-plated on blood agar plates, and the plates were incubated (Infors HT Multitron, UK) overnight at 37 °C to enumerate colony-forming units in the inoculum. Two hundred microlitres of MHB containing an inoculum with 3×10^5^ c.f.u. per well and different concentrations of the test antibiotics were added to each well in a 96-well plate. A concentration of antibiotics ranging from 0.006 to 32 mg l^−1^ was tested. The MIC value was determined as the lowest concentration of an antibiotic which completely inhibits visible bacterial growth after 24 h at 37 °C in static conditions. In total six antibiotics were tested: gentamicin, meropenem and ciprofloxacin. The optical density at 600 nm was measured using the TECAN Spark plate reader (TECAN, Switzerland) to confirm the growth inhibition. One column of each 96-well plate was designated for growth control and one for sterility control. The procedure was repeated three times across different days for each antibiotic.

A stock solution of gentamicin sulphate was prepared by dissolving 0.1 g of antibiotic in 10 ml of sterile distilled water. Ciprofloxacin hydrochloride was dissolved in PBS, pH 6.0 to the final stock concentration of 25000 mg l^−1^. Meropenem trihydrate was dissolved in one part of methanol mixed with nine parts of PBS to the final stock concentration of 1000 mg l^−1^. Stock solutions were stored in aliquots of 2 ml at −20^o^ C, used promptly on defrosting and unused leftover solutions were discarded.

### Biofilm susceptibility, equivalence and MBEC assays

Biofilm susceptibility testing was conducted using a Calgary device (Innovotech, Canada), with the biofilm grown on a peg, as outlined by Harrison *et al*. [50]. We initially performed an equivalence test for biofilm formation to ensure consistent growth conditions (Fig. S1, available in the online version of this article), following the methodology described previously [50]. The test specifically examined whether there was a variation in the number of bacteria retrieved from pegs between columns and rows in the 96-well plate, thereby ensuring uniform and comparable results across all wells.

The bacterial strains were streaked out on an LB agar plate from cryogenic stock and incubated overnight at 37 °C. A single colony from the agar subculture was used to inoculate 5 ml Mueller–Hinton Broth (MHB) cation-adjusted and the suspension was incubated in a 50 ml Falcon tube while shaking at 110 r.p.m. for 24 h at 37 °C (Infors HT Multitron, UK). The bacterial suspension was centrifuged at 4000 ***g*** in Eppendorf 5710R (Thermo Fisher, UK) for 5 min. After discarding the supernatant, the pellet was resuspended in 5 ml of sterile MHB. The inoculum size was prepared in a fresh centrifuge tube by diluting the suspension of bacteria ten times to an optical density (OD) of 0.05 at 600 nm. The OD_600 nm_ was measured using the spectrophotometer Jenway (VWR, UK). The inoculum was pipetted in a 96-well plate with a final concentration of 8×10^6^ c.f.u. of *P. aeruginosa* PA01 or PA14 per well (0.15 ml inoculum in each well). One column in a 96-well plate was used as a control and contained media without bacteria added. Pegs from the Calgary Device were immersed in the inoculum. The 96-well plate was double sealed with parafilm, placed inside a plastic box to reduce media evaporation and incubated (statically) overnight at 37 °C with 70 % humidity in the incubator (Infors HT Multitron, UK) to allow biofilm formation on pegs. Before each experiment, 0.01 ml of sixfold dilutions of the inoculum were spot-plated on blood agar plates, and the plates were incubated overnight at 37 °C to enumerate c.f.u. in the inoculum. After overnight incubation, the pegs were rinsed twice for 1 min in two 96-well plates with 0.2 ml of sterile water per well to remove bacteria that did not attach to the pegs (planktonic cells).

For the equivalence assay, the pegs were then transferred to a 96-well plate with 0.2 ml of LB with 1 % Tween 20 per well, sonicated for 10 min at 60 Hz to disrupt bacteria from the biofilm on pegs into a recovery medium. After sonication, 0.02 ml of the MHB cation-adjusted media with the bacteria was diluted in a series up to 10^4^ in 0.18 ml of sterile water. All dilutions were plated out on LB agar plates for c.f.u. count and incubated at 37 °C overnight (Fig. S1).

For the MBEC assay, the pegs were transferred after rinsing steps to a 96-well plate with antibiotics in MHB. The plate was incubated overnight and then rinsed and sonicated in the same way as equivalence assay plates. Ciprofloxacin, meropenem and gentamicin were tested with concentrations starting from 1 mg l^−1^ to 512 mg l^−1^. The MBEC was determined as the wells with the lowest concentration of an antibiotic where the biofilm was completely eradicated, i.e. there was no growth from biofilms across all replicates. One column of each 96-well plate was designated for untreated control and one for sterility control. The procedure was repeated four times across different days for each antibiotic with four technical replicates each time.

### Testing antibiotics on an *ex vivo* porcine cornea model

In this study, porcine eyes were extracted within 4 h of slaughter and transported from the abattoir (R.B. Elliott and Son Abattoir, Calow, England) in a Nalgene container filled with sterile phosphate-buffered saline (PBS, Sigma, Germany). The age of the pigs ranged between 26 to 28 weeks. Importantly, it is essential to note that the pigs were sacrificed for human consumption and not for this study. The extraction, incubation and infection procedures were followed as published previously [34] with some improvements. Subsequently, corneas with 2 mm of surrounding sclera were excised in the laboratory within 2 h of delivery and incubated in a combination of Dulbecco’s Modified Eagle’s Medium (DMEM) and Ham’s F12 Nutrient Mixture (1 : 1) with antibiotics in a six-well plate for 24 h [34]. Following this, the corneas were washed twice with 1 ml PBS and incubated in an antimicrobial-free DMEM: Ham’s F12 media for 48 h to remove residual antibiotics from the previous media. Throughout this time, the medium was replaced daily. We confirmed the absence of bacterial growth inhibition after 48 h in media without antibiotics (unpublished data). Additionally, we regularly inspected corneas for contamination by processing control (uninfected) corneas and incubating a small sample of media from each cornea on LB agar just before infection experiments.

Initially, the infection timeline was optimized and the outcomes were assessed at 1, 2, 4, 6 and 24 h. Before every experiment, the bacteria were incubated in LB broth at 37 °C with 150 r.p.m. shaking for 4 h. The culture was centrifuged at 4000*** g*** for 5 min, and the supernatant was discarded. The pellet was resuspended (washed) with PBS and centrifuged as described above three times. The pellet was resuspended in PBS to the desired optical density. On the infection day, porcine corneas were wounded four times with a scalpel, placed inside the glass moulds and mounted on the agar as described previously [34]. The 10 mm diameter of the central corneal surface within a glass mould was exposed to an inoculum of about 8×10^6^ c.f.u. in 0.2 ml of PBS. After a 6 h incubation in a six-well dish, the PBS, along with the suspended bacteria were removed using a sterile 1 ml pipette tip. Subsequently, it was replaced either with 0.2 ml of PBS for control corneas or PBS supplemented with antibiotics for treated corneas. The corneas were treated with either 1024 mg l^−1^ or, as outlined in Table 1, the MIC concentration of ciprofloxacin, meropenem and gentamicin for 18 h at 37 °C.

To capture the effects of the treatments, all corneas were photographed (Figs S2 and S3) with a Dino-lite Xcope camera (AnMo Electronics Corporation, Taiwan).

### Statistics

The microbial reduction was calculated according to the following formula: Bacteria reduction percentage (%) = (control c.f.u. – test c.f.u.)/control c.f.u.)×100. Statistical analysis comparing the effect of treatment versus treatment control was calculated using the Kruskal–Wallis multiple comparisons test, while the input c.f.u. between groups was compared using one-way ANOVA, using GraphPad Prism version 8.4.1. *P*-values<0.05 were considered significant.

## Results

### Antibiotic sensitivity in MIC assay

MIC assays were conducted to assess the sensitivity of two strains of *P. aeruginosa* to gentamicin, meropenem and ciprofloxacin (Table 1). Both strains exhibited sensitivity to tested antibiotics (Table 1). While the MIC values for gentamicin were consistent between strains (2–4 mg l^−1^), marginal differences were observed in their susceptibility to meropenem and ciprofloxacin. PA14 showed marginally higher susceptibility to meropenem (0.25 mg l^−1^) while PA01 exhibited marginally higher susceptibility to ciprofloxacin (0.125–0.25 mg l^−1^).

**Table 1.** Determination of MIC and MBC of *P. aeruginosa* for invasive PA01 and cytotoxic PA14 strains against gentamicin, meropenem and ciprofloxacin. Values in the table represent mg l^−1^

Generic name(class)Break points [49]	PA14		PA01		Mechanism of action
	MIC	MBEC	MIC	MBEC	
Gentamicin(aminoglycoside)≤4 s; ≥16 (R)	2–4	16(4X - 8X MIC)	2–4	64(16X - 32X MIC)	Broad spectrum, inhibits synthesis of bacterial proteins by binding to 30S ribosomes
Meropenem(carbapenem)≤2 (S); ≥8 (R)	0.25	>512	0.5–1	>512	Broad spectrum, inhibition of bacterial cell wall synthesis
Ciprofloxacin(fluoroquinolone)≤0.001 (S); ≥0.5 (R)	0.25–0.5	4(8X - 16X MIC)	0.125–0.25	4–8(16X - 64X MIC)	Inhibits DNA replication by inhibiting bacterial DNA topoisomerase and DNA-gyrase

Despite demonstrating sensitivity to meropenem in MIC assays (Table 1), biofilm formed by both *P. aeruginosa* strains exhibited tolerance to meropenem concentrations exceeding those tested (>512 mg l^−1^). For the invasive PA01 strain, MBEC values were 16–64 times higher than the MIC for ciprofloxacin and 16–32 times higher than MIC for gentamicin (Table 1). Similarly, MBEC values for the cytotoxic PA14 strain were 8–16 times higher than MIC for ciprofloxacin and 4–8 times higher than the MIC for gentamicin. These findings indicate that biofilms formed by the cytotoxic strain PA14 were comparatively more susceptible to gentamicin and ciprofloxacin compared to the invasive strain PA01 (Table 1). It is worth noting that there was one log less c.f.u.s retrieved from pegs for PA14 compared to PA01 (Fig. S1) in MBEC assays, which could have influenced drug efficacy. Additionally, this data suggests that in the same conditions, PA01 forms thicker biofilms than PA14. The MBEC testing accentuated the differences between the two strains.

With reference to the breakpoint system outlined in Table 1 and subsequent clinical relevance, the MBEC results suggest that biofilms formed by *P. aeruginosa* could be classified as tolerant to gentamicin, meropenem and ciprofloxacin.

### Effect of inoculum size on final bacterial load

**Fig. 1.**
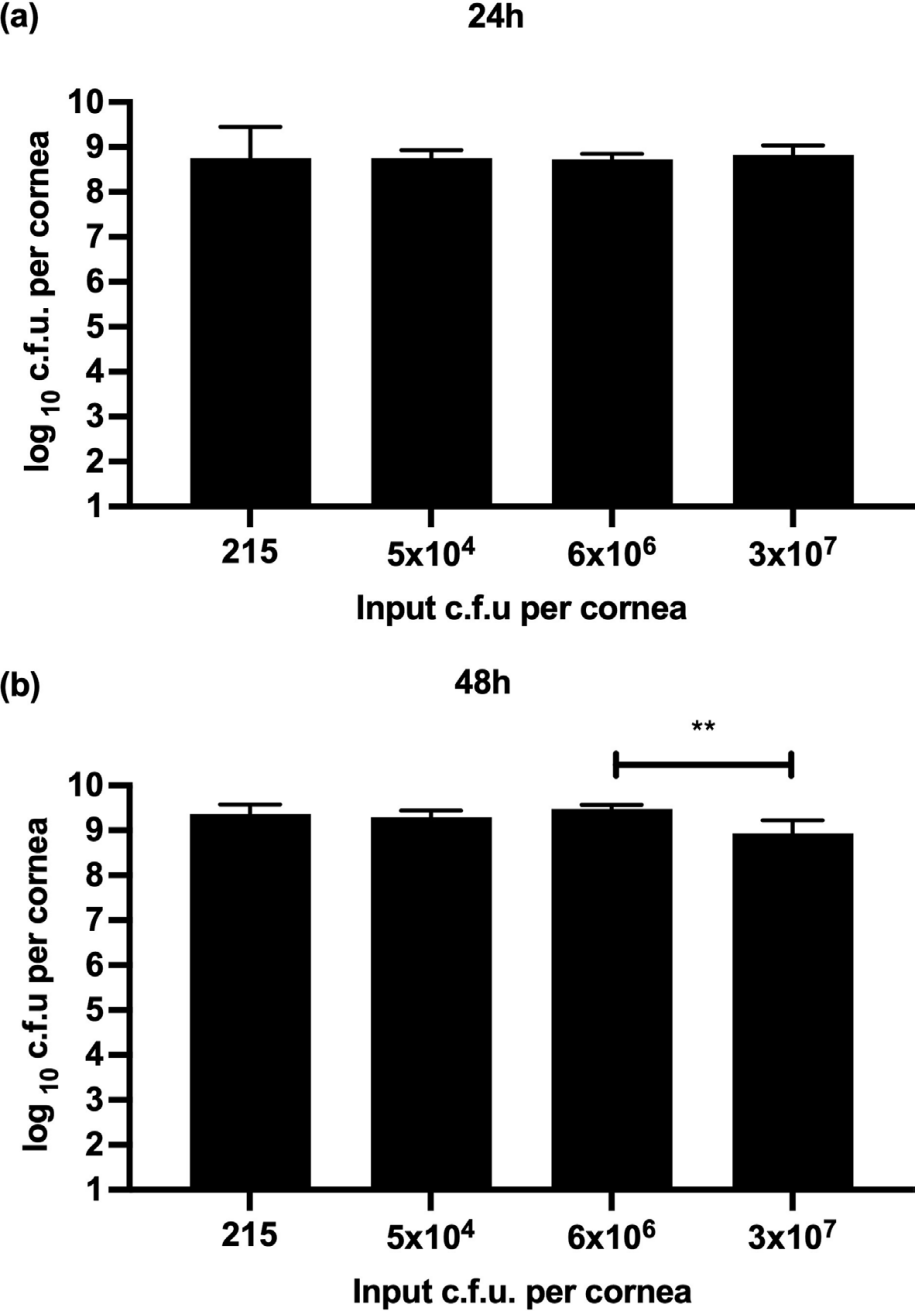
Number of viable *P. aeruginosa* PA14 retrieved from porcine cornea after infection with 215, 5×10^4^, 6×10^6^ and 3×10^7^ c.f.u. per cornea. Corneas were infected for 24 h (*n*=28) (**a**) and 48 h (*n*=29) (**b**). Bars show geometric means with 95 % confidence indicated by error bars. Statistical significance of the difference between c.f.u. inputs was calculated according to the one-way ANOVA test. Unless otherwise labelled, no significant difference was observed. *P*-values: *<0.05; **<0.005.

To establish the inoculum size needed to initiate an infection in the porcine cornea, various c.f.u.s of *P. aeruginosa* PA14 were added to wounded corneas. A viable count of bacteria retrieved from the infected cornea after 24 h of infection (*n*=28) (Fig. 1a) and 48 h of infection (*n*=29) (Fig. 1b) was carried out. These experiments were carried out before introducing washing steps. Despite the starting inoculum size, an average of 6×10^8^ c.f.u.s per cornea were retrieved after 24 h and 2×10^9^ c.f.u.s per cornea after 48 h. Most c.f.u. counts showed no significant difference across groups and incubation times, except for the two groups with the highest initial c.f.u. after 48 h. This discrepancy may stem from complete corneal lysis in the highest input group, resulting in a sticky homogenate that hindered accurate pipetting and potentially led to an underestimated c.f.u. count. These results indicate that the ultimate bacterial load in the porcine *ex vivo* cornea infection model is independent of the initial bacterial load. Due to the good reproducibility in the number of c.f.u. retrieved after infection with a higher starting inoculum size, in further experiments, an inoculum size of greater than 1×10^6^ c.f.u. per cornea was aimed for. We established that the maximum incubation time for all following experiments was 24 h because 48 h of incubation resulted in complete lysis of the cornea by the bacteria.

### Effect of incubation time on the progress of infection

**Fig. 2.**
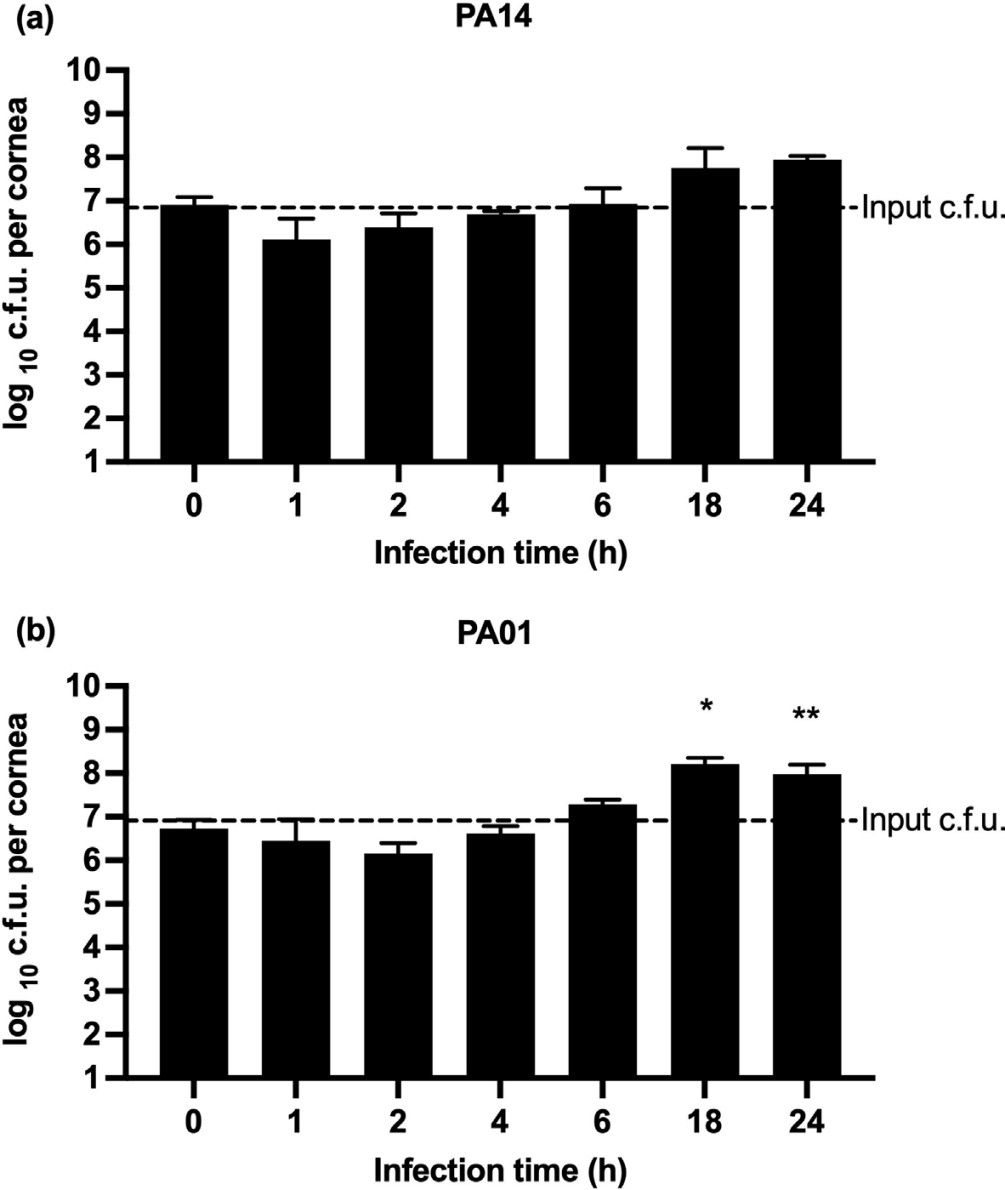
Increase in c.f.u. of *P. aeruginosa* with time in *ex vivo* porcine corneas. Corneas were infected for 1, 2, 4, 6, 18 and 24 h with *P. aeruginosa* PA14 (*n*=41) (**a**) and *P. aeruginosa* PA01 (*n*=67) (**b**). Actual inoculum c.f.u. are shown both as c.f.u. at 0 h infection time as well as a dotted line labelled Input c.f.u. Bars show geometric means with 95 % confidence indicated by error bars. Statistical significance of the difference from input c.f.u. was calculated according to the Kruskall–Wallis test. Unless otherwise labelled, no significant difference was observed. *P*-values: *<0.05; **<0.005.

To investigate the progress of infection over time, porcine corneas were infected with *P. aeruginosa* PA14 and *P. aeruginosa* PA01 and a viable count was carried out on bacteria retrieved from the infected cornea after 1, 2, 4, 6, 18 and 24 h post-infection (h p.i.) (Fig. 2). With *P. aeruginosa* PA14, an average of 1.9×10^6^ c.f.u. per cornea were retrieved after 1 h p.i. (*n*=7), 2.9×10^6^ c.f.u. per cornea were retrieved after 2 h p.i. (*n*=6), and 4.9×10^6^ c.f.u. per cornea were retrieved after 4 h p.i. (*n*=6) (Fig. 2a). At all these time points, the number of c.f.u. retrieved per cornea were lower than the inoculum size (7.7×10^6^ c.f.u. per cornea) reflecting the impact of post-incubation rinsing steps included in the protocol during which the bacterial population not securely adhered to the corneal tissue are removed. After 6 h p.i., the number of bacteria retrieved from the infected cornea was approximately equal to the inoculum size despite rinsing (*n*=6). Incubation beyond 6 h p.i. reproducibly resulted in a clear increase of c.f.u. retrieved per cornea despite rinsing, resulting in 1.0×10^8^ c.f.u. per cornea at 18 h p.i. (*n*=6) and 9.0×10 ^7^ at 24 h p.i. (*n*=6) (Fig. 2a). Difference in c.f.u. values for PA14 retrieved at 1 h p.i. and 2 h p.i. in comparison to 18 h p.i. and 24 h p.i. was significant (*P*<0.05).

A similar trend was seen in the progress of infection in the *ex vivo* porcine cornea infected with *P. aeruginosa* PA01 strain (Fig. 2b). An average of 3.4×10^6^ c.f.u. per cornea were retrieved after 1 h p.i. (*n*=4), 2.2×10^6^ c.f.u. per cornea were retrieved after 2 h p.i. (*n*=14) and 4.1×10^6^ c.f.u. per cornea were retrieved at 4 h p.i. (*n*=6). Like the infection with *P. aeruginosa* PA14, at all these time points, the number of c.f.u. retrieved per cornea was lower than the inoculum size (7.9×10^6^ c.f.u. per cornea). Subsequently, the increase in bacteria load in the infected cornea was higher compared to the inoculum size for *P. aeruginosa* PA01 (Fig. 2b): 2.0×10^7^ c.f.u. per cornea at 6 h p.i. (*n*=6), 1.6×10^8^ c.f.u. per cornea at 18 h p.i. (*n*=4) and 1.7×10^8^ c.f.u. per cornea at 24 h p.i. (*n*=25) (Fig. 2b). Difference in c.f.u. values for PA01 retrieved at 1 h p.i., 2 h p.i. and 4 h p.i. in comparison to 18 h p.i. and 24 h p.i. was significant (*P*<0.05).

These data demonstrate that both strains of *P. aeruginosa* were able to initiate and maintain infection on porcine corneas within the first few hours of incubation. In both strains, despite the inclusion of a washing step, there was a net increase in the number of c.f.u. retrieved after incubation compared to the inoculum, which suggests that infection was well established in the model. In the subsequent experiments, antibiotic treatments were added to corneas at 6 h p.i. because there was a visible increase in c.f.u. counts at this time point in comparison to the input of bacteria which indicated that the infection was well-established.

## Investigation of antimicrobial efficacy on the *ex vivo* porcine keratitis model

### Testing MIC concentrations of antibiotics on the *ex vivo* porcine keratitis model

**Fig. 3.**
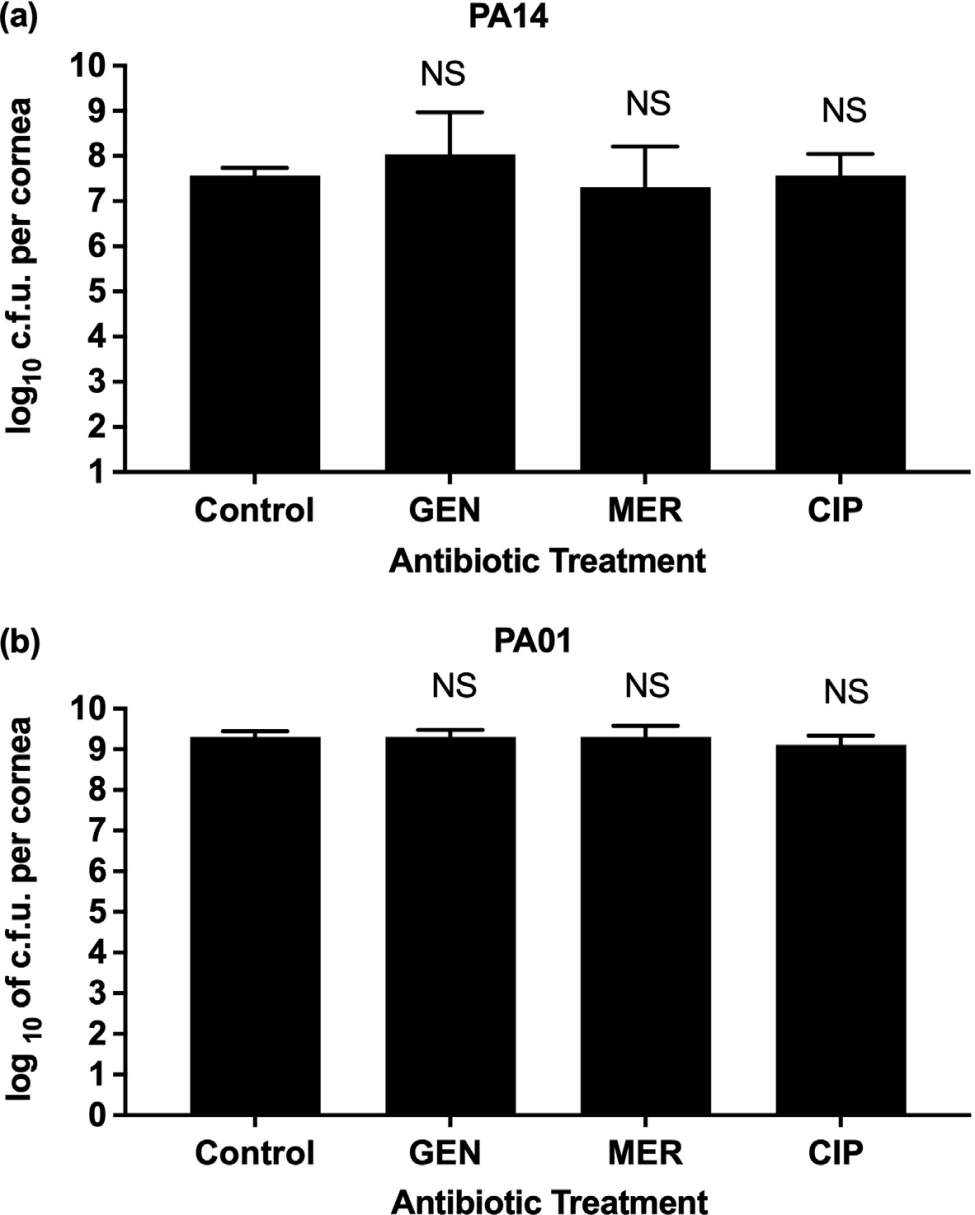
Effects of different antibiotics at MIC on c.f.u. of *P. aeruginosa* in ex vivo porcine corneas. Corneas were infected for 6 h with (**a**) PA14 or (**b**) PA01. Control corneas were immersed in PBS while other corneas were treated with MIC concentrations of antibiotics gentamicin (GEN) (*n*=4 for PA14 and *n*=4 for PA01), meropenem (MER) (*n*=4) and ciprofloxacin (CIP) (*n*=4) dissolved in PBS. Bars show geometric means with 95 % confidence indicated by error bars. Statistical significance of the difference from untreated controls was calculated according to the Kruskall–Wallis test. *P*-values: NS >0.05.

Firstly, the effect of MIC concentrations of antibiotics on infected tissue was investigated and imaged (Fig. S2). *Ex vivo* porcine corneas were infected on average with 1×10^7^ c.f.u. *P*. *aeruginosa* PA14 (*n*=16) and 9×10^6^ *P. aeruginosa* PA01 (*n*=15) for 6 h and then MIC concentrations of gentamicin, meropenem and ciprofloxacin were applied for 18 h. While MIC concentrations of antibiotics successfully inhibited the growth of bacteria *in vitro*, these concentrations were ineffective (*P*>0.05) for both tested strains of *P. aeruginosa* PA14 and PA01 in the *ex vivo* porcine cornea model (Fig. 3 & Table S1). This demonstrates that the application of MIC concentrations of these antibiotics on *ex vivo* cornea is insufficient to inhibit the growth of * P. aeruginosa* even though the infected tissue was continually exposed to the antibiotic for 18 h.

### Testing 1024 mg l^−1^ concentrations of antibiotics on the *ex vivo* porcine keratitis model

**Fig. 4.**
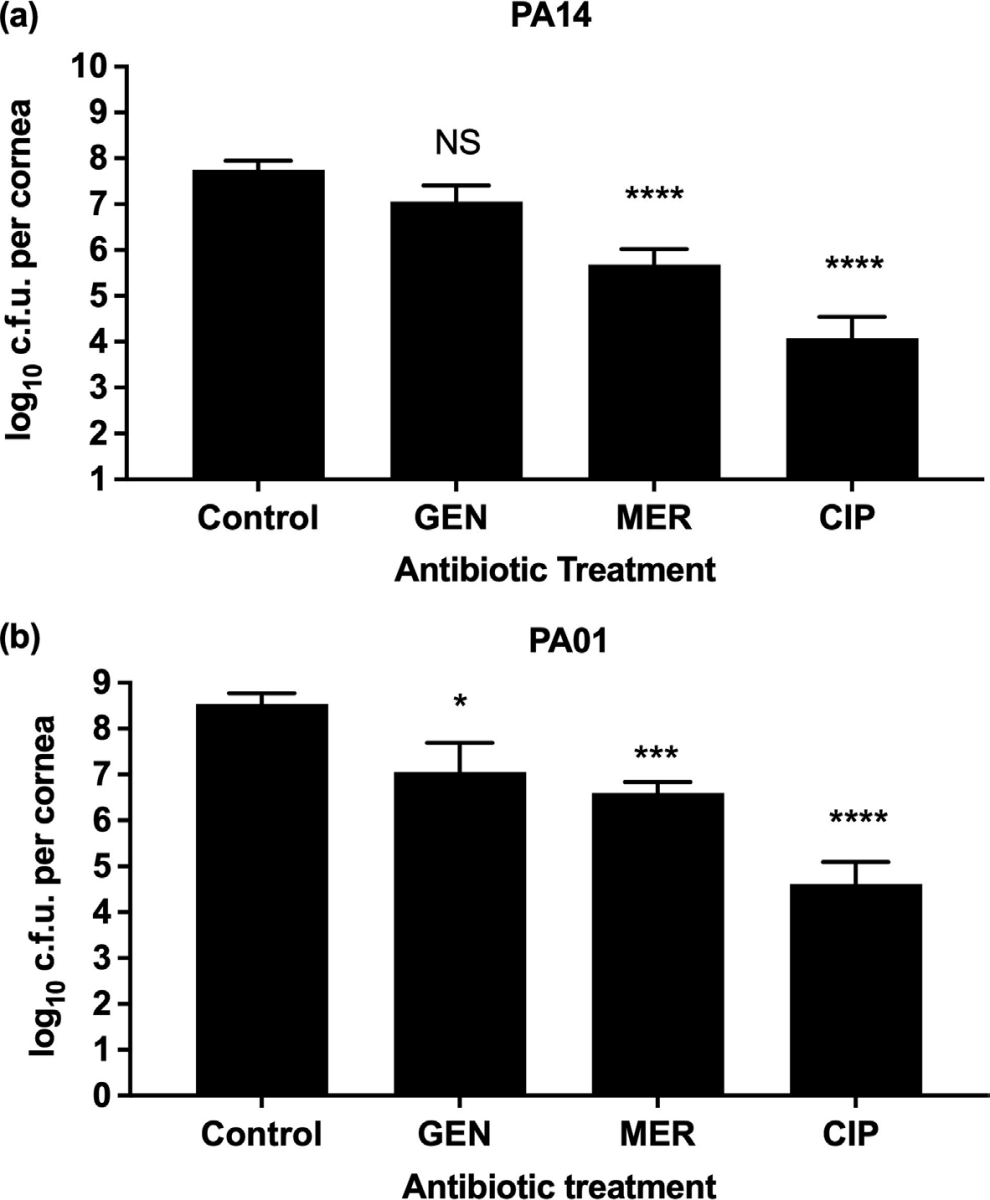
Effects of different antibiotics at supra-MIC on c.f.u. of *P. aeruginosa* in ex vivo porcine corneas Corneas were infected for 6 h with (**a**) PA14 or (**b**) PA01. Control corneas (*n*=35) were immersed in PBS while other corneas were treated with supra-MIC (1024 mg l^−1^) of antibiotics gentamicin (GEN) (*n*=12), meropenem (MER) (*n*=12) and ciprofloxacin (CIP) (*n*=12) dissolved in PBS. Bars show geometric means with 95 % confidence indicated by error bars. Statistical significance of the difference from untreated controls was calculated according to the Kruskall-Wallis test. *P*-values: NS >0.05; *<0.05 ***<0.005 ****<0.0005.

The concentration of antibiotics (gentamicin, meropenem and ciprofloxacin) that were applied on *ex vivo* porcine corneas was increased to 1024 mg l^−1^ (Fig. 4). This concentration is 256 times MIC for gentamicin for strains PA01 and PA14, respectively. For meropenem, this concentration is 1025 times MIC for invasive strain PA01 and 4100 times MIC meropenem for cytotoxic PA14. For ciprofloxacin, this concentration is 4100 times MIC for strain PA01 and 2050 times MIC for strain PA14. As this concentration is higher than MIC (and MBEC) some growth inhibition and improved transparency on *ex vivo* infected tissue was expected (Fig. S3). A significant reduction in bacteria load for invasive strain PA01 in corneas treated with gentamicin (*n*=12, *P*=0.0051), meropenem (*n*=12, *P*<0.0001) and ciprofloxacin (*n*=12, *P*<0.0001) was observed when compared to controls (Fig. 4 & Table S2). In contrast, there was no significant reduction for corneas infected with cytotoxic strain PA14 and treated with gentamicin (*n*=12, *P*=0.15) whereas treatment of PA14 with meropenem (*n*=12, *P*=0.0001) and ciprofloxacin (*n*=12, *P*<0.0001) had a noticeable reduction in bacteria load. However, none of the antibiotics eradicate bacteria at tested concentrations.

## Discussion

In our previous work [34], we established an *ex vivo* porcine model of *Pseudomonas aeruginosa* keratitis, providing a platform for testing treatments against corneal infections. Here, we investigated the efficacy of gentamicin, meropenem and ciprofloxacin against *P. aeruginosa* strains PA14 and PA01 in our model. The corneas were cut with a scalpel aiming to increase the tissue penetration of bacteria and applied treatment. The treatment was applied continuously to increase the antibiotic penetration of the tissue at the infection site.

We first established MIC and MBEC values for gentamicin, meropenem and ciprofloxacin using cytotoxic strain PA14 and invasive strain PA01 of *P. aeruginosa*. Next, we monitored the development of an infection over time. Finally, we investigated differences in response to antibiotic treatments between cytotoxic strain PA14 and invasive strain PA01 of *P. aeruginosa* on the *ex vivo* porcine keratitis model.

The comparison of MIC and MBEC values to literature was complicated by variances in experimental protocols among research groups [23,51]. Nevertheless, our findings were consistent with literature trends, indicating the need for higher antibiotic concentrations to eradicate antimicrobial-resistant strains compared to planktonic bacteria [52,55]. The lower bacteria count for PA14 in the equivalence test suggests that the biofilm formed by this strain was likely thinner compared to PA01. More robust biofilms bind bacteria firmly into the matrix, increasing the bacteria’s survival [56]. This disparity in biofilm thickness combined with variations in gene expression linked to biofilm-specific antibiotic resistance [52, 55] may have contributed to differences in MBEC between strains.

In this study gentamicin exhibited efficacy against both strains, with PA01 demonstrating slightly higher tolerance in MBEC testing, aligning with prior observations [28,53, 55]. These results indicate that while gentamicin is effective against planktonic *P. aeruginosa*, higher concentrations are needed to treat biofilm [54]. The tolerance of *P. aeruginosa* biofilm toward gentamicin could be explained by the fact that gentamicin belongs to the aminoglycosides group of antibiotics known to bind to various components in the biofilm matrix [29], such as exopolysaccharides Psl [28] and Pel [30], which would impede the biofilm penetration and the drug efficacy.

Meropenem is known for its excellent corneal penetration and low cytotoxicity [57]. It is unknown if it penetrates corneal cells. MIC values for *P. aeruginosa* PA01 showed sensitivity to meropenem, consistent with literature findings ranging from 0.1 to 0.5 mg l^−1^ [42,53, 58, 59]. Similarly, the MIC values for cytotoxic strain PA14 aligned with previous studies at 0.25 mg l^−1^ [59]. However, MBEC values consistently surpassed MIC values in the literature, indicating reduced efficacy against established biofilm [42,53]. Moreover, PA01 can develop rapid meropenem tolerance in the biofilms [42]. These findings suggest that meropenem is more effective against actively dividing, planktonic bacteria or early-stage biofilms, but less effective against established biofilms [42,53].

Finally, we investigated ciprofloxacin, a highly efficacious treatment for the *P. aeruginosa* keratitis [39]. Our study confirmed its potency against both planktonic and biofilm forms of *P. aeruginosa in vitro* and *ex vivo* cornea models with PA01 slightly more tolerant than PA14. MIC values indicated susceptibility in both strains, with higher tolerance towards antibiotics in biofilms, consistent with existing literature [45,53, 60,63]. Similarly, our MBEC results mirrored previous findings, revealing minimal differences in ciprofloxacin response between cytotoxic PA14 and invasive PA01 biofilms [28,64].

In our initial studies aimed at establishing an *ex vivo* keratitis model, we experimented with varying bacterial loads in the inoculum to initiate infection. We found that as few as 215 c.f.u. of *P. aeruginosa* PA14 per cornea were sufficient to initiate infection in our *ex vivo* model and the colony counts retrieved from the cornea after 24 h remained consistent regardless of the initial inoculum size. We hypothesize that this stabilisation at 24 h post-infection may be attributed to nutrient limitation, leading bacteria to enter a stationary growth phase, akin to batch cultures [65,66]. In the literature, researchers often use inoculum loads equal to or greater than 1×10^6^ c.f.u. of *Pseudomonas sp*. per eye *in vivo* [36,67, 68]. Furthermore, as previously noted [33], the lack of standardized protocol for *ex vivo* studies precludes direct comparison of our results with others. Considering that a higher inoculum facilitates reliable bacterial quantification we opted for an inoculum containing at least 1×10^6^ c.f.u. per cornea in subsequent experiments.

To discern whether infections caused by cytotoxic and invasive strains of *P. aeruginosa* could be distinguished, we monitored the progression of infection over time by comparing the c.f.u. counts retrieved from the cornea infected with cytotoxic *P. aeruginosa* PA14 and invasive *P. aeruginosa* PA01 strains. Interestingly, we observed growth plateaus after 18 h of incubation with both strains, indicating that bacteria entered a stationary phase at this point. Heightened cytotoxicity of *P. aeruginosa* PA14 did not appear to confer a selective advantage during infection of the wounded *ex vivo* porcine cornea. Consequently, we concluded that the enhanced cytotoxicity did not significantly influence the progression of infection in our porcine keratitis model. The efficacy of antibiotic treatment was assessed by evaluating colony-forming units obtained from each cornea. It is important to acknowledge that viable but not culturable cells (VBNC) were not considered and this fact could pose a limitation to this study [69]. Alternative methods for validating bacterial numbers may be investigated in future research. Nevertheless, it is worth mentioning that the number of retrieved colonies was consistent across all corneas and the results were reproducible.

Finally, when the antibiotics were tested on the *ex vivo* keratitis model, we found that gentamicin was ineffective in both strains at MIC concentrations of 2 and 4 mg l^−1^. Even at concentrations higher than MBEC of 1024 mg l^−1^ efficacy was poor in our *ex vivo* keratitis model. Studies on rabbits *in vivo* used various, usually much higher than ours, concentrations of gentamicin (1600, 3000, 5000 and 13 000 mg l^−1^) to treat *P. aeruginos*a keratitis, yielding mixed therapeutic outcomes [70,73]. It is challenging to directly compare our results from infection treatment outcomes to *in vivo* because of differences in experimental setups [33]. Although we did not measure if gentamicin reached MIC in tissue, this antibiotic shows good corneal tissue penetration; therefore, the concentration of this antibiotic more likely reached MIC values [74]. Additionally, wounding corneas in our study created a defect that is expected to increase the penetration of an antibiotic [75]. Also, gentamicin demonstrates a post-antibiotic effect (PAE), where bacteria growth is inhibited following exposure, even after the drug concentration has fallen below MIC [76]. According to the literature, cytotoxic strains of *P. aeruginosa* (PA14) remain mainly outside the host cells, while invasive strains (PA01) reside and replicate inside corneal cells during infection. Therefore, it is believed that antibiotics that do penetrate host-cell membranes, such as tobramycin or gentamicin, are often less effective against invasive strains of *P. aeruginosa*, while ofloxacin (e.g. ciprofloxacin) that penetrate host cell membranes can be used to target these strains [36,77].

Meropenem, characterised by low toxicity and excellent corneal tissue penetration [57] has shown remarkable efficacy in treating *Pseudomonas* keratitis at concentrations of 50000 mg l^−1^ in both rabbit [78,79] and human studies [7], without any observed side effects. Studies have indicated that meropenem concentrations of 5000 mg l^−1^ can enhance cellular activity in corneal epithelial cell lines, with high cell viability (96 %) post-treatment [57]. These findings suggest that meropenem can be used at high concentrations without toxic side effects. Additionally, some studies show that meropenem may be a viable option, particularly when *P. aeruginosa* strains exhibit tolerance to ciprofloxacin or gentamicin [7]. In our *ex vivo* cornea experiments, despite the much lower concentration used compared to *in vivo* studies, meropenem demonstrated a significant reduction in bacterial load, consistent with findings in *ex vivo* rabbit and human studies [78,79]. Despite meropenem’s efficacy, concerns arise from *P. aeruginosa* tolerance in the MBEC data [42,53]. Notably, Haagensen *et al*. [42] showed meropenem’s high effectiveness in the early stages of *P. aeruginosa* PA01 biofilm formation.

Our study demonstrated a reduction in bacterial load after 1024 mg l^−1^ of meropenem application within 6 h post-infection, possibly targeting the early stages of biofilm formation. However, the dose used was much lower than used in clinical settings, insufficient to complete bacterial clearance. Further studies are needed to assess meropenem’s effectiveness and its potential in clinical application. Additionally, studies suggest a synergistic effect when combining meropenem and ciprofloxacin against certain clinical isolates of * P. aeruginosa* [80,82], a combination that could be explored in our *ex vivo* porcine keratitis model in the future.

Ciprofloxacin exhibits good tissue and cell penetration properties, with studies demonstrating that even brief exposure of as little as 10 min can lead to concentrations surpassing the MIC in human corneas *ex vivo* [75,83, 84]. The factors that contribute to ciprofloxacin’s high permeability include the low molecular mass and lipophilicity, allowing crossing through the hydrophobic corneal epithelium [85]. Given the 18 h continuous exposure in our study, it is highly likely that ciprofloxacin reached MIC concentrations in corneal tissue. However, it was discovered that only 10 % of the measured ciprofloxacin levels in a chemical assay were bioavailable [85], which may explain the lack of bacterial growth inhibition in corneas exposed to MIC concentrations. Our experiments revealed that ciprofloxacin was the most potent in inhibiting the growth of *P. aeruginosa* at higher concentrations. Although the concentrations of ciprofloxacin applied in this study were insufficient to eradicate the bacteria it is likely because in clinical practice much higher than MIC and MBEC concentrations are used [23]. Also, we hypothesize that ciprofloxacin’s ability to penetrate corneal cells and eradicate bacteria internally [46] was an important factor in its effectiveness. Additionally, the treatment demonstrated slightly higher efficacy in the PA14 strain compared to PA01, albeit insignificantly [36], despite the fact that cytotoxic strains remain extracellular, while invasive strains penetrate corneal cells. Numerous studies have shown that ciprofloxacin significantly reduces or completely halts *P. aeruginosa* infection in live rabbits [86,87] and humans [88]. However, it was found that phenotypic adaptation towards persistence to ciprofloxacin occurs early if supra-MIC concentrations are used, potentially leading to failure in eradicating biofilms [45].

Despite both meropenem and ciprofloxacin demonstrating sensitivity in MIC testing, their effectiveness against biofilm in MBEC was diminished. Even though being more potent than gentamicin, none of the tested antibiotics eradicated bacteria in our *ex vivo Pseudomonas* keratitis model, even with continuous exposure to antibiotics. The increased potency of ciprofloxacin in our keratitis model may be attributed to its ability to penetrate cells, amongst many other factors, thereby targeting bacteria both extracellularly and intracellularly, as demonstrated in primary human corneal fibroblasts *in vitro* [46]. This could explain the slightly higher reduction observed after ciprofloxacin and meropenem treatment in *ex vivo* corneas infected with the cytotoxic PA14 strain, which remains extracellular, compared to the invasive PA01 strain, which additionally infiltrates corneal cells and replicates internally. Gentamicin, akin to tobramycin, lacks cell permeability, likely contributing to its poor efficacy. In contrast, ciprofloxacin’s ability to penetrate cells explains its superior effectiveness compared to gentamicin. Meropenem, with a smaller molecular mass than gentamicin and only slightly larger than ciprofloxacin, shares a similar trend, prompting further investigation into its cell permeability. Furthermore, our *ex vivo* keratitis model showed no significant difference in antimicrobial response to ciprofloxacin and gentamicin between cytotoxic and invasive strains of *P. aeruginosa*, aligning with previous *in vivo* studies [36].

The present study has several limitations that require consideration. Firstly, our *ex vivo* model lacks fully operating host defences, including infiltrating immune cells, nerves, tear fluid, and blinking, which are essential components in the defence against infection and influence outcomes. The absence of these factors may not only affect the state of the host tissue but also the localization and environment of the bacteria, potentially altering bacterial gene expression in response to their surroundings. Although there is evidence supporting tissue viability and epithelial healing in cultured corneas *ex vivo* in the literature [89,90], the 3 day storage period may introduce variability and deviate from *in vivo* conditions due to lack of host factors and slower healing process. Moreover, the presence of opacity after incubation and before infection highlights the need to consider the dynamic changes that occur in corneal tissue over time post-excision. However, we demonstrated previously that the infection outcome was the same regardless of the presence or absence of swelling [34]. Furthermore, we used viable c.f.u. counts to assess the bacteria load after antibiotic treatment. It is well established that bacteria may be viable but non-culturable after antibiotic treatment. Thus, only using c.f.u. counting may miss a population of bacteria that is viable in the biofilm and the tissue. The model does not account for certain tear components, which might influence bacterial susceptibility to antibiotics [91] or inhibit bacterial growth [92]. However, given the early stage of our investigation, we opted to maintain simplicity in the model design and therefore used PBS, like in studies *in vivo* on mice [36]. Further validation of this model is required. Despite these limitations, our study’s response to antibiotic treatment aligns with trends found in the literature, suggesting that our *ex vivo* keratitis model shares similarities with other animal models *in vivo* and clinical studies on humans. Therefore, while acknowledging these limitations, we propose that our *ex vivo* porcine cornea model could be a valuable tool for rapidly and cost-effectively screening the efficacy of ocular antibiotics with good sensitivity and reliability, alongside *in vitro* studies.

## supplementary material

10.1099/mic.0.001459 Uncited Supplementary Material 1.

## Abbreviations

c.f.u.: colony forming units
CIP: ciprofloxacin
DMEM: Dulbecco’s Modified Eagle’s Medium
DNA: deoxyribonucleic acid
EUCAST: European Committee on Antimicrobial Susceptibility Testing
GEN: gentamicin
h: hours
h p.i.: hours post infection
LB: Lysogeny Broth
MBEC: minimum biofilm eradication concentration
MER: meropenem
mg l^-1^: milligram per liter
MHB: Mueller-Hinton Broth
MIC: minimum inhibitory concentration
min: minutes
ml: millilitre
n: number of replicates
^o^C: degrees celcius
OD: optical density
p: probability
PAE: post-antibiotic effect
*P. aeruginosa*: *Pseudomonas aeruginosa*
PBS: phosphate buffered saline
pH: potential of hydrogen ions
VBNC: viable but not culturable cells

## References

[1] Humphries RM, Abbott AN, Hindler JA (2019). Understanding and addressing CLSI breakpoint revisions: a primer for clinical laboratories. J Clin Microbiol.

[2] Moussa G, Hodson J, Gooch N, Virdee J, Penaloza C (2021). Calculating the economic burden of presumed microbial keratitis admissions at a tertiary referral centre in the UK. Eye.

[3] Whitcher J, Srinivasan M, Upadhyay M (2001). Corneal blindness: a global perspective. Bull World Health Organ.

[4] Ung L, Bispo PJM, Shanbhag SS, Gilmore MS, Chodosh J (2019). The persistent dilemma of microbial keratitis: global burden, diagnosis, and antimicrobial resistance. Surv Ophthalmol.

[5] Garg P, Sharma S, Rao GN (1999). Ciprofloxacin-resistant *Pseudomonas keratitis*. Ophthalmology.

[6] Behlau I, Gilmore MS (2008). Microbial biofilms in ophthalmology and infectious disease. Arch Ophthalmol.

[7] Elhardt C, Wolf A, Wertheimer CM (2023). Successful treatment of multidrug-resistant *Pseudomonas aeruginosa* keratitis with meropenem eye drops - a case report. J Ophthalmic Inflamm Infect.

[8] Radford R, Brahma A, Armstrong M, Tullo AB (2000). Severe sclerokeratitis due to *Pseudomonas aeruginosa* in noncontact-lens wearers. Eye.

[9] Shoji MK, Gutkind NE, Meyer BI, Yusuf R, Sengillo JD (2023). Multidrug-resistant *Pseudomonas aeruginosa* keratitis associated with artificial tear use. JAMA Ophthalmol.

[10] Thi MTT, Wibowo D, Rehm BHA (2020). *Pseudomonas aeruginosa* Biofilms. Int J Mol Sci.

[11] Moledina M, Roberts HW, Mukherjee A, Spokes D, Pimenides D (2023). Analysis of microbial keratitis incidence, isolates and in-vitro antimicrobial susceptibility in the East of England: a 6-year study. Eye.

[12] Khan M, Ma K, Wan I, Willcox MDP (2023). Ciprofloxacin resistance and tolerance of *Pseudomonas aeruginosa* ocular isolates. Cont Lens Anterior Eye.

[13] López-Dupla M, Martínez JA, Vidal F, Almela M, Soriano A (2009). Previous ciprofloxacin exposure is associated with resistance to beta-lactam antibiotics in subsequent *Pseudomonas aeruginosa* bacteremic isolates. Am J Infect Control.

[14] O’Brien TP (2003). Management of bacterial keratitis: beyond exorcism towards consideration of organism and host factors. Eye.

[15] Willcox MDP (2011). Review of resistance of ocular isolates of *Pseudomonas aeruginosa* and *staphylococci* from keratitis to ciprofloxacin, gentamicin and cephalosporins. Clin Exp Optom.

[16] Dalmon C, Porco TC, Lietman TM, Prajna NV, Prajna L (2012). The clinical differentiation of bacterial and fungal keratitis: a photographic survey. Invest Ophthalmol Vis Sci.

[17] Ibrahim YW, Boase DL, Cree IA (2009). Epidemiological characteristics, predisposing factors and microbiological profiles of infectious corneal ulcers: the Portsmouth corneal ulcer study. Br J Ophthalmol.

[18] Norina TJ, Raihan S, Bakiah S, Ezanee M, Liza-Sharmini AT (2008). Microbial keratitis: aetiological diagnosis and clinical features in patients admitted to hospital universiti Sains Malaysia. Singapore Med J.

[19] Varaprasathan G, Miller K, Lietman T, Whitcher JP, Cevallos V (2004). Trends in the etiology of infectious corneal ulcers at the F. I. Proctor Foundation. Cornea.

[20] Kaye S (2017). Microbial keratitis and the selection of topical antimicrobials. BMJ Open Ophthalmol.

[21] Tuft S, Somerville TF, Li JPO, Neal T, De S (2022). Bacterial keratitis: identifying the areas of clinical uncertainty. Prog Retin Eye Res.

[22] Gokhale NS (2008). Medical management approach to infectious keratitis. Indian J Ophthalmol.

[23] Kowalska-Krochmal B, Dudek-Wicher R (2021). The minimum inhibitory concentration of antibiotics: methods, interpretation, clinical relevance. Pathogens.

[24] Herbert R, Caddick M, Somerville T, McLean K, Herwitker S (2022). Potential new fluoroquinolone treatments for suspected bacterial keratitis. BMJ Open Ophthalmol.

[25] Davies D (2003). Understanding biofilm resistance to antibacterial agents. Nat Rev Drug Discov.

[26] Lebeaux D, Ghigo JM, Beloin C (2014). Biofilm-related infections: bridging the gap between clinical management and fundamental aspects of recalcitrance toward antibiotics. Microbiol Mol Biol Rev.

[27] Hall CW, Mah TF (2017). Molecular mechanisms of biofilm-based antibiotic resistance and tolerance in pathogenic bacteria. FEMS Microbiol Rev.

[28] Billings N, Millan MR, Caldara M, Rusconi R, Tarasova Y (2013). The extracellular matrix component Psl provides fast-acting antibiotic defense in *Pseudomonas aeruginosa* biofilms. PLoS Pathog.

[29] Ciofu O, Tolker-Nielsen T (2019). Tolerance and resistance of *Pseudomonas aeruginosa* biofilms to antimicrobial agents-how *P. aeruginosa* can escape antibiotics. Front Microbiol.

[30] Colvin KM, Gordon VD, Murakami K, Borlee BR, Wozniak DJ (2011). The pel polysaccharide can serve a structural and protective role in the biofilm matrix of *Pseudomonas aeruginosa*. PLoS Pathog.

[31] Jacobs HM, O’Neal L, Lopatto E, Wozniak DJ, Bjarnsholt T (2022). Mucoid *Pseudomonas aeruginosa* can produce calcium-gelled biofilms independent of the matrix components Psl and CdrA. J Bacteriol.

[32] Kumar NG, Nieto V, Kroken AR, Jedel E, Grosser MR (2022). *Pseudomonas aeruginosa* can diversify after host cell invasion to establish multiple intracellular niches. mBio.

[33] Urwin L, Okurowska K, Crowther G, Roy S, Garg P (2020). Corneal infection models: tools to investigate the role of biofilms in bacterial keratitis. Cells.

[34] Okurowska K, Roy S, Thokala P, Partridge L, Garg P (2020). Establishing a porcine ex vivo cornea model for studying drug treatments against bacterial keratitis. J Vis Exp.

[35] Bonanno JA (2012). Molecular mechanisms underlying the corneal endothelial pump. Exp Eye Res.

[36] Lee EJ, Truong TN, Mendoza MN, Fleiszig SMJ (2003). A comparison of invasive and cytotoxic *Pseudomonas aeruginosa* strain-induced corneal disease responses to therapeutics. Curr Eye Res.

[37] Wozniak DJ, Wyckoff TJO, Starkey M, Keyser R, Azadi P (2003). Alginate is not a significant component of the extracellular polysaccharide matrix of PA14 and PAO1 Pseudomonas aeruginosa biofilms. Proc Natl Acad Sci U S A.

[38] Freschi L, Vincent AT, Jeukens J, Emond-Rheault JG, Kukavica-Ibrulj I (2019). The *Pseudomonas aeruginosa* pan-genome provides new insights on its population structure, horizontal gene transfer, and pathogenicity. Genome Biol Evol.

[39] Hilliam Y, Kaye S, Winstanley C (2020). *Pseudomonas aeruginosa* and microbial keratitis. J Med Microbiol.

[40] Borkar DS, Fleiszig SMJ, Leong C, Lalitha P, Srinivasan M (2013). Association between cytotoxic and invasive *Pseudomonas aeruginosa* and clinical outcomes in bacterial keratitis. JAMA Ophthalmol.

[41] Goodman AL, Kulasekara B, Rietsch A, Boyd D, Smith RS (2004). A signaling network reciprocally regulates genes associated with acute infection and chronic persistence in *Pseudomonas aeruginosa*. Dev Cell.

[42] Haagensen J, Verotta D, Huang L, Engel J, Spormann AM (2017). Spatiotemporal pharmacodynamics of meropenem- and tobramycin-treated *Pseudomonas aeruginosa* biofilms. J Antimicrob Chemother.

[43] Kasetty S, Katharios-Lanwermeyer S, O’Toole GA, Nadell CD (2021). Differential surface competition and biofilm invasion strategies of *Pseudomonas aeruginosa* PA14 and PAO1. J Bacteriol.

[44] Mikkelsen H, McMullan R, Filloux A (2011). The *Pseudomonas aeruginosa* reference strain PA14 displays increased virulence due to a mutation in ladS. PLoS One.

[45] Soares A, Roussel V, Pestel-Caron M, Barreau M, Caron F (2019). Understanding ciprofloxacin failure in *Pseudomonas aeruginosa* biofilm: persister cells survive matrix disruption. Front Microbiol.

[46] Cendra MD, Christodoulides M, Hossain P (2017). Effect of different antibiotic chemotherapies on *Pseudomonas aeruginosa* infection *in vitro* of primary human corneal fibroblast cells. Front Microbiol.

[47] EUCAST (2003). Determination of minimum inhibitory concentrations (MICs) of antibacterial agents by broth dilution. Clin Microbiol Infect.

[48] Hasselmann C (2000). European society of clinical microbiology and infectious diseases (ESCMID) - European Committee for antimicrobial susceptibility testing (EUCAST) - determination of minimum inhibitory concentration (MIC) by Agar dilution. Clin Microbiol Infect.

[49] EUCAST (2022).

[50] Harrison JJ, Stremick CA, Turner RJ, Allan ND, Olson ME (2010). Microtiter susceptibility testing of microbes growing on peg lids: a miniaturized biofilm model for high-throughput screening. Nat Protoc.

[51] Schuurmans JM, Hayali ASN, Koenders BB, ter Kuile BH (2009). Variations in MIC value caused by differences in experimental protocol. J Microbiol Methods.

[52] Bagge N, Schuster M, Hentzer M, Ciofu O, Givskov M (2004). *Pseudomonas aeruginosa* biofilms exposed to imipenem exhibit changes in global gene expression and beta-lactamase and alginate production. Antimicrob Agents Chemother.

[53] Bowler LL, Zhanel GG, Ball TB, Saward LL (2012). Mature *Pseudomonas aeruginosa* biofilms prevail compared to young biofilms in the presence of ceftazidime. Antimicrob Agents Chemother.

[54] Mah T-F, Pitts B, Pellock B, Walker GC, Stewart PS (2003). A genetic basis for *Pseudomonas aeruginosa* biofilm antibiotic resistance. Nature.

[55] Zhang L, Fritsch M, Hammond L, Landreville R, Slatculescu C (2013). Identification of genes involved in *Pseudomonas aeruginosa* biofilm-specific resistance to antibiotics. PLoS One.

[56] Sutherland IW (2001). Biofilm exopolysaccharides: a strong and sticky framework. Microbiology.

[57] Sueke H, Kaye S, Wilkinson MC, Kennedy S, Kearns V (2015). Pharmacokinetics of meropenem for use in bacterial keratitis. Invest Ophthalmol Vis Sci.

[58] Monahan LG, Turnbull L, Osvath SR, Birch D, Charles IG (2014). Rapid conversion of *Pseudomonas aeruginosa* to a spherical cell morphotype facilitates tolerance to carbapenems and penicillins but increases susceptibility to antimicrobial peptides. Antimicrob Agents Chemother.

[59] Ocampo-Sosa AA, Cabot G, Rodríguez C, Roman E, Tubau F (2012). Alterations of OprD in carbapenem-intermediate and -susceptible strains of *Pseudomonas aeruginosa* isolated from patients with bacteremia in a Spanish multicenter study. Antimicrob Agents Chemother.

[60] Bruchmann S, Dötsch A, Nouri B, Chaberny IF, Häussler S (2013). Quantitative contributions of target alteration and decreased drug accumulation to *Pseudomonas aeruginosa* fluoroquinolone resistance. Antimicrob Agents Chemother.

[61] Fernández-Olmos A, García-Castillo M, Maiz L, Lamas A, Baquero F (2012). In vitro prevention of *Pseudomonas aeruginosa* early biofilm formation with antibiotics used in cystic fibrosis patients. Int J Antimicrob Agents.

[62] Riera E, Macià MD, Mena A, Mulet X, Pérez JL (2010). Anti-biofilm and resistance suppression activities of CXA-101 against chronic respiratory infection phenotypes of *Pseudomonas aeruginosa* strain PAO1. J Antimicrob Chemother.

[63] Shafiei M, Ali AA, Shahcheraghi F, Saboora A, Akbari Noghabi K (2014). Eradication of *Pseudomonas aeruginosa* biofilms using the combination of n-butanolic cyclamen coum extract and ciprofloxacin. Jundishapur J Microbiol.

[64] Benthall G, Touzel RE, Hind CK, Titball RW, Sutton JM (2015). Evaluation of antibiotic efficacy against infections caused by planktonic or biofilm cultures of *Pseudomonas aeruginosa* and *Klebsiella pneumoniae* in Galleria mellonella. Int J Antimicrob Agents.

[65] Llorens JMN, Tormo A, Martínez-García E (2010). Stationary phase in gram-negative bacteria. FEMS Microbiol Rev.

[66] Rolfe MD, Rice CJ, Lucchini S, Pin C, Thompson A (2012). Lag phase is a distinct growth phase that prepares bacteria for exponential growth and involves transient metal accumulation. J Bacteriol.

[67] Augustin DK, Heimer SR, Tam C, Li WY, Le Due JM (2011). Role of defensins in corneal epithelial barrier function against *Pseudomonas aeruginosa* traversal. Infect Immun.

[68] Tam C, LeDue J, Mun JJ, Herzmark P, Robey EA (2011). 3D quantitative imaging of unprocessed live tissue reveals epithelial defense against bacterial adhesion and subsequent traversal requires MyD88. PLoS One.

[69] Li L, Mendis N, Trigui H, Oliver JD, Faucher SP (2014). The importance of the viable but non-culturable state in human bacterial pathogens. Front Microbiol.

[70] Frucht-Pery J, Golan G, Hemo I, Zauberman H, Shapiro M (1995). Efficacy of topical gentamicin treatment after 193-nm photorefractive keratectomy in an experimental *Pseudomonas keratitis* model. Graefe’s Arch Clin Exp Ophthalmol.

[71] Kowalski RP, Kowalski TA, Shanks RMQ, Romanowski EG, Karenchak LM (2013). In vitro comparison of combination and monotherapy for the empiric and optimal coverage of bacterial keratitis based on incidence of infection. Cornea.

[72] Punitan R, Sulaiman SA, Hasan HB, Shatriah I (2019). Clinical and antibacterial effects of Tualang honey on Pseudomonas-induced keratitis in rabbit eyes. Cureus.

[73] Rootman DS, Krajden M (1993). Continuous flow perfusion of gentamicin with a scleral shell reduces bacterial colony counts in experimental *Pseudomonas keratitis*. J Ocul Pharmacol.

[74] Wilhelmus KR, Abshire RL (2003). Corneal ciprofloxacin precipitation during bacterial keratitis. Am J Ophthalmol.

[75] McDermott ML, Tran TD, Cowden JW, Buggé CJL (1993). Corneal stromal penetration of topical ciprofloxacin in humans. Ophthalmology.

[76] Karlowsky JA, Zhanel GG, Davidson RJ, Hoban DJ (1994). Postantibiotic effect in *Pseudomonas aeruginosa* following single and multiple aminoglycoside exposures in vitro. J Antimicrob Chemother.

[77] Fleiszig SMJ, Zaidi TS, Pier GB (1995). *Pseudomonas aeruginosa* invasion of and multiplication within corneal epithelial cells in vitro. Infect Immun.

[78] Bozkurt E, Muhafiz E, Kepenek HS, Bozlak ÇEB, Koç Saltan S (2021). A new treatment experience in *Pseudomonas keratitis*: topical meropenem and cefepime. Antimicrob Agents Chemother.

[79] Pandey RA, Bhailume PV (2014). Use of topical meropenem in management of hospital acquired Pseudomonas ocular infections. J Clin Ophthalmol.

[80] Erdem I, Kaynar-Tascioglu J, Kaya B, Goktas P (2002). The comparison of in the vitro effect of imipenem or meropenem combined with ciprofloxacin or levofloxacin against multidrug-resistant *Pseudomonas aeruginosa* strains. Int J Antimicrob Agents.

[81] Pankuch GA, Lin GR, Seifert H, Appelbaum PC (2008). Activity of meropenem with and without ciprofloxacin and colistin against *Pseudomonas aeruginosa* and *Acinetobacter baumannii*. Antimicrob Agents Chemother.

[82] Siqueira VLD, Cardoso RF, Caleffi-Ferracioli KR, Scodro RBD, Fernandez MA (2014). Structural changes and differentially expressed genes in *Pseudomonas aeruginosa* exposed to meropenem-ciprofloxacin combination. Antimicrob Agents Chemother.

[83] Oztürk F, Kortunay S, Kurt E, Ilker SS, Basci NE (1999). Penetration of topical and oral ciprofloxacin into the aqueous and vitreous humor in inflamed eyes. Retina.

[84] Silva GCM, Jabor VAP, Bonato PS, Martinez EZ, Faria-E-Sousa SJ (2017). Penetration of 0.3% ciprofloxacin, 0.3% ofloxacin, and 0.5% moxifloxacin into the cornea and aqueous humor of enucleated human eyes. Braz J Med Biol Res.

[85] Kaye SB, Neal T, Nicholson S, Szkurlat J, Bamber S (2009). Concentration and bioavailability of ciprofloxacin and teicoplanin in the cornea. Invest Ophthalmol Vis Sci.

[86] Bu P, Riske PS, Zaya NE, Carey R, Bouchard CS (2007). A comparison of topical chlorhexidine, ciprofloxacin, and fortified tobramycin/cefazolin in rabbit models of *Staphylococcus* and *Pseudomonas keratitis*. J Ocul Pharmacol Ther.

[87] LaBorwit SE, Katz HR, Hirschbein MJ, Oswald MR, Snyder LS (2001). Topical 0.3% ciprofloxacin vs topical 0.3% ofloxacin in early treatment of *Pseudomonas aeruginosa* keratitis in a rabbit model. Ann Ophthalmol.

[88] Levey SB, Katz HR, Levine ES, Abrams DA, Marsh MJ (1998). Efficacy of topical ciprofloxacin 0.3% in the treatment of ulcerative keratitis in humans. Ann Ophthalmol Glaucoma.

[89] Castro N, Gillespie SR, Bernstein AM (2019). Ex vivo corneal organ culture model for wound healing studies. J Vis Exp.

[90] Schumann S, Dietrich E, Kruse C, Grisanti S, Ranjbar M (2021). Establishment of a robust and simple corneal organ culture model to monitor wound healing. J Clin Med.

[91] Sebbag L, Broadbent VL, Kenne DE, Perrin AL, Mochel JP (2021). Albumin in tears modulates bacterial susceptibility to topical antibiotics in ophthalmology. Front Med.

[92] Fleiszig SMJ, Kwong MSF, Evans DJ (2003). Modification of *Pseudomonas aeruginosa* interactions with corneal epithelial cells by human tear fluid. Infect Immun.

