# Supplementary material for: Increased tolerance to commonly used antibiotics in a Pseudomonas aeruginosa ex vivo porcine keratitis model
Source: Microbiology (Reading). 2024 May 13;170(5):001459. doi: 10.1099/mic.0.001459 (PMC11165664; doi:10.1099/mic.0.001459)
Supplement: Uncited Supplementary Material 1. [file mic-170-01459-s001.pdf]

Supplementary materials

Equivalence assay

Before initiating MBEC assays, we optimised experimental conditions using an equivalence assay to ensure uniformity and comparability of experimental conditions across different wells or columns within a 96-well plate (n = 8 pegs per column). It aimed to investigate if the biofilm growth was similar between pegs across columns. The variance in c.f.u. between columns was insignificant for both strains ($p > 0.05$), which confirmed the equivalence between experimental conditions. However, we observed about 1 log less c.f.u. of the PA14 strain (Fig. S1a) attached to pegs compared to PA01 (Fig. S1b) which suggests that PA14 biofilms were thinner (Fig. S1). This could have accentuated the differences between both strains in the MBEC assay treated with antibiotics.

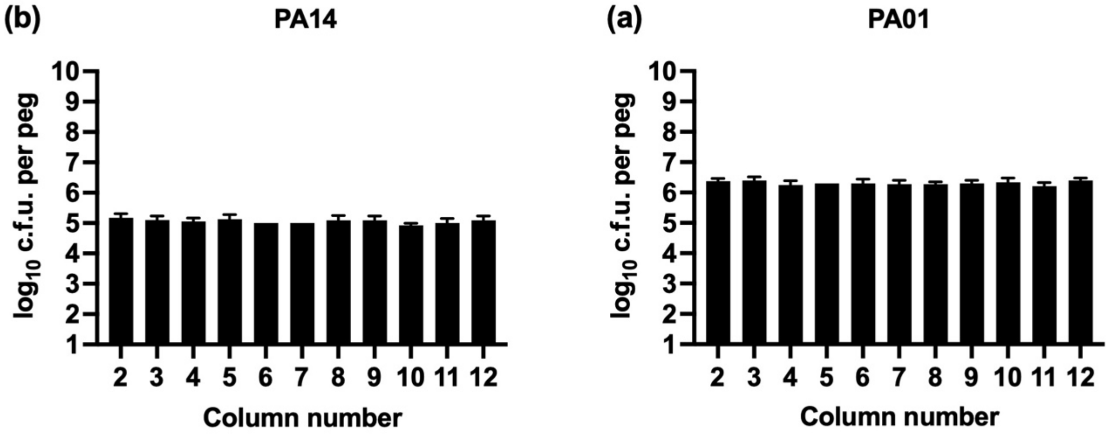

Fig. S1. Equivalence assay results representing colony-forming units of *P. aeruginosa* PA14 (a) and PA01 (b) retrieved from pegs (n = 8 pegs per column) across all columns in a 96-well plate. Bars show geometric means with 95% confidence indicated by standard deviation bars. The statistical significance of the difference from c.f.u. between pegs was calculated according to the one-way ANOVA test. Unless otherwise labelled, no significant difference was observed. *p*-values: *<0.05; **<0.005.

Investigation of antimicrobial efficacy on the *ex vivo* porcine keratitis model

Imaging infected *ex vivo* porcine corneas treated with MIC concentrations of antibiotics

All infected and treated corneas were photographed before homogenisation (Fig. S2 & S3). *Ex vivo* corneas often swell slightly when kept in media for several days, resulting in a slightly hazy appearance (as observed in uninfected corneas, Fig. S2 & S3). Clinically, *P. aeruginosa* keratitis typically presents with a large epithelial defect associated with stromal necrosis,

characterised by a ring-like, milky-coloured stromal infiltrate. The manifestation of *P. aeruginosa* infection on *ex vivo* porcine cornea closely resembled the features observed in clinical infections *in vivo* (Fig. S2 & S3 PBS). Visually, there was a reduction in white discolouration on all corneas treated with MIC concentration of gentamicin, meropenem and ciprofloxacin compared to untreated infected corneas (PBS). This difference was more pronounced in corneas infected with the PA14 strain (Fig. S2A). Despite no reduction in viable c.f.u. across all antibiotics (Fig. 3), corneas infected with both *P. aeruginosa* strains and treated with MIC concentration of gentamicin appeared slightly hazier compared to meropenem and ciprofloxacin.

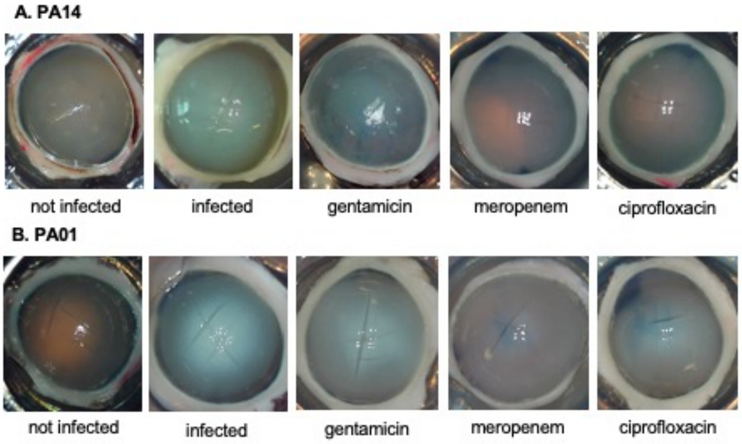

Fig. S2. Representative images of non-infected and infected *ex vivo* porcine corneas without treatment (PBS) and treated with gentamicin, meropenem and ciprofloxacin. The corneas shown here were infected with $6x10^6$ c.f.u. of strain PA14 (A) and strain PA01 (B) and treated with MIC concentrations of antibiotics after the infection had progressed for 6 hours. Corneas were imaged and immediately homogenised for viable counting.

Imaging infected *ex vivo* porcine corneas treated with 1024 mg L^{-1} concentrations of antibiotics

Treatment with a higher antibiotic concentration (1024 mg L^{-1}) decreased corneal opacity in infected corneas by preventing the development of a milky colour (Fig. S3). This effect was particularly pronounced in corneas infected with the PA14 strain. Corneas treated with gentamicin, meropenem and ciprofloxacin appeared clear and visually indistinguishable from uninfected corneas, indicating a direct effect of treatment on opacity (Fig. S3). Despite the high bacterial count (Fig. 4), gentamicin treatment preserved corneal transparency.

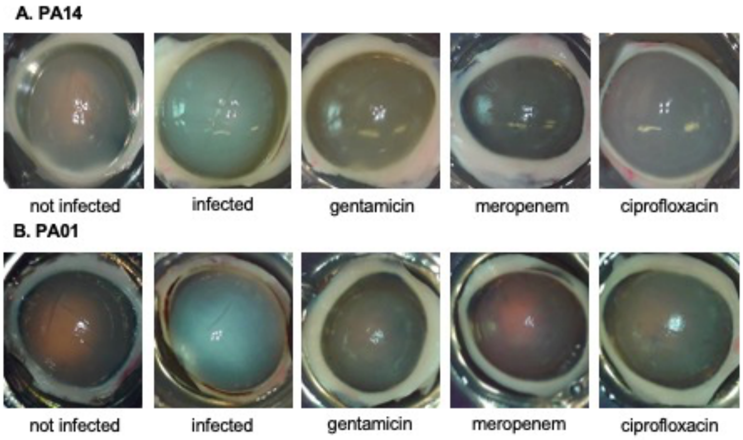

Fig. S3. Representative images of non-infected and infected *ex vivo* porcine corneas without treatment (PBS) and treated with 1024 mg L^{-1} of gentamicin, meropenem and ciprofloxacin. The corneas shown here were infected with $6x10^6$ c.f.u. of strain PA14 (A) and strain PA01 (B) 6 hours before antibiotic treatment. Corneas were imaged and immediately homogenised for viable counting.

Testing MIC concentrations of antibiotics on the *ex vivo* porcine keratitis model

Table S1. Data summary of average colony forming units of *P. aeruginosa* in the *ex vivo* porcine corneas infected for 6 hours with PA14 or PA01 and treated with MIC concentrations of gentamicin, ciprofloxacin and meropenem.

PA14

Treatment	Concentration mg L^{-1}	Average c.f.u.	SD	N	% Reduction
PBS	0	4.E+07	1.E+07	4	
Gentamicin	4	2.E+08	2.E+08	4	0.000
Ciprofloxacin	0.5	3.E+07	2.E+07	4	15.302
Meropenem	0.25	5.E+07	5.E+07	4	0.000

PA01

Treatment	Concentration mg L^{-1}	Average	SD	N	% Reduction
PBS	0	3.E+09	8.E+08	4	
Gentamicin	4	2.E+09	8.E+08	4	26.005
Ciprofloxacin	0.5	2.E+09	1.E+09	4	43.625
Meropenem	1	1.E+09	9.E+08	4	53.651

Testing 1024 mg L^{-1} concentrations of antibiotics on the *ex vivo* porcine keratitis model

Table S2. Data summary of average colony forming units of *P. aeruginosa* in the *ex vivo* porcine corneas infected for 6 hours with PA14 or PA01 and treated with 1024 mg L^{-1} gentamicin, ciprofloxacin and meropenem.

PA14

Treatment	Concentration mg L^{-1}	Average c.f.u.	SD	N	% Reduction	Log Reduction
PBS	0	1.E+08	6.E+07	16		
Gentamicin	1.024	2.E+07	2.E+07	12	79.266	< 1 log
Ciprofloxacin	1.024	5.E+04	2.E+04	12	99.955	3 log
Meropenem	1.024	9.E+05	1.E+06	12	99.125	2 log

PA01

Treatment	Concentration mg L^{-1}	Average c.f.u.	SD	N	% Reduction	Log Reduction
PBS	0	8.E+08	2.E+09	19		
Gentamicin	1.024	7.E+07	1.E+08	12	91.260	1 log
Ciprofloxacin	1.024	1.E+05	1.E+05	12	99.985	3 log
Meropenem	1.024	5.E+06	4.E+06	12	99.340	2 log
